# The Impact of Vascular Disease Treatment on Platelet-Derived Microvesicles

**DOI:** 10.1007/s10557-017-6757-7

**Published:** 2017-11-22

**Authors:** Justyna Rosińska, Maria Łukasik, Wojciech Kozubski

**Affiliations:** Department of Neurology, Poznan University of Medical Sciences, ul. Przybyszewskiego 49, 60-355 Poznan, Poland

**Keywords:** Platelet-derived microvesicles (pMV), Platelet-derived microparticles, Antiplatelet therapy, Statins, Cerebrovascular disease, Cardiovascular disease

## Abstract

Platelet-derived microvesicles (pMVs) are small, heterogeneous vesicles released from platelet membranes as a result of activation. These microvesicles possess a wide range of properties, including prothrombotic, proatherogenic, proinflammatory, immunomodulatory, and even anticoagulant activity. The elevated release of these microvesicles has been observed in various metabolic, inflammatory, thrombotic, and vascular diseases, including ischemic heart disease, stroke, hypertension, diabetes, and connective tissue disease. Modulation of both pMV generation and the expression of their surface molecules may have beneficial clinical implications and could become a novel therapeutic target. However, mechanisms by which pharmacological agents can modify pMV formation are elusive. The purpose of this review is to discuss the effects of drugs routinely used in primary and secondary prevention of vascular disease on the release of pMV and expression of their surface procoagulant and proinflammatory molecules.

## Introduction

**Fig. 1:**
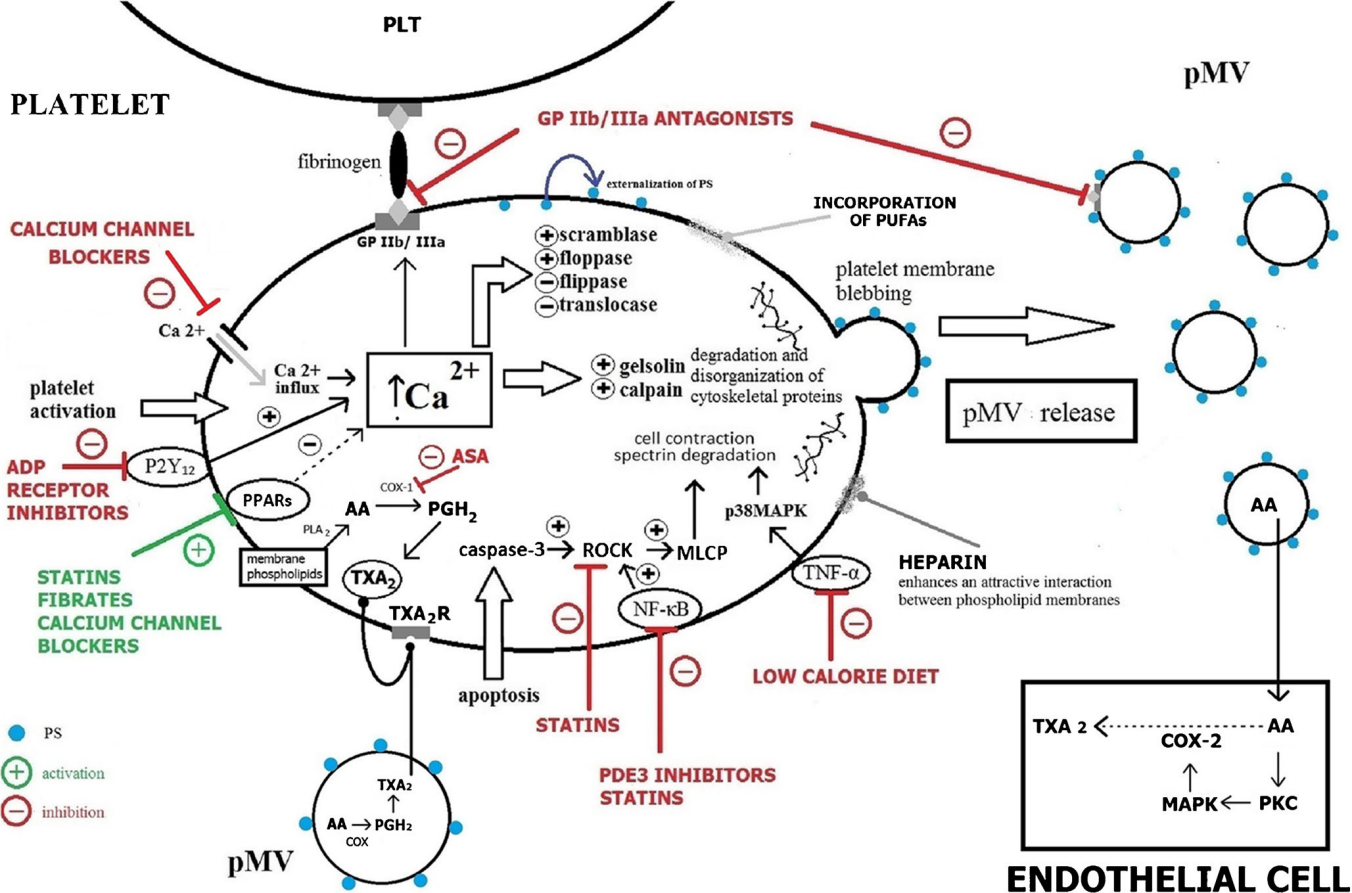
Potential effects of vascular disease treatment on pMV release. Increase in intraplatelet calcium concentration is the principal step in pMV formation. ADP receptor inhibitors increase the intraplatelet concentration of cAMP thereby decreasing platelet vesiculation. GP IIb-IIIa antagonists inhibit binding of fibrinogen thereby preventing the second wave of platelet activation. Statins inhibit platelet vesiculation multi-directional—reducing NF-κB activity and increasing exposure of PPARs and via the ROCK pathway. Fibrates as PPAR agonists increase the levels of both cAMP and cGMP and decrease calcium concentration. Calcium channel blockers inhibit calcium influx and decrease intracellular calcium concentration. Platelet-derived microvesicles transfer AA between platelets and ECs. Microvesicles also metabolize AA to TXA2. AA arachidonic acid, ADP adenosine diphosphate, ASA acetylsalicylic acid, COX cyclooxygenase, GP glycoprotein, MLCP myosin light chain phosphatise, MAPK mitogen-activated protein kinase, NF-κB nuclear factor kappa B, PDE phosphodiestherase, PGH2 prostaglandin H2, PKC protein kinase C, PLA2 phospholipase A2, PLT platelet, p38MAPK mitogen-activated protein kinase p38, pMV platelet-derived microvesicles, PPAR peroxisome proliferator-activated receptor, PS phosphatidylserine, PUFAs polyunsaturated fatty acids, ROCK Rho-associated protein kinase, TNF-α tumor necrosis factor α, TXA2 thromboxane A2, TXA2R thromboxane A2 receptor

Platelet-derived microvesicles (pMV) are small (0.1–1.0 μm in diameter), heterogeneous vesicles released from platelet membranes as a result of activation following cytoskeletal changes and externalization of the negatively charged inner layer phospholipids. Microvesicles (MVs) of platelet origin, first described in 1967 by Wolf et al. as “platelet dust” [1], being the most abundant and accounting for approximately 70–90% of all circulating MV [2, 3], are currently the best recognized. Although the role of pMV has been overlooked and underestimated for a long time, and they have even been treated as debris, pMVs have recently attracted substantial attention. Circulating pMVs have been widely reported in plasma from healthy individuals [3, 4]. However, elevated concentrations have been observed in various conditions associated with platelet activation such as ischemic stroke [5–8], coronary artery disease (CAD) [9–11], hypertension [12], diabetes [13], obesity [14, 15], cancer, and metastases [16, 17], suggesting a potential correlation between the quantity of MV and the clinical severity of the disease. Therefore, modulation of pMV generation as well as the exposure of their surface markers may have beneficial clinical implications and become novel therapeutic targets in the treatment of various pathological conditions (Table 1). The number of studies on the impact of drugs on pMV is limited, and their results are equivocal. The research is related mainly to the effect of therapeutic agents used in the prevention of cardiovascular disease (CVD), such as antiplatelet, antihypertensive, and hypolipidemic agents. Most reports are from clinical studies, and little information is available on the mechanisms of action of drugs on pMV release (Fig. 1). Understanding these mechanisms has fundamental clinical and therapeutic implications, especially in vascular diseases.

**Table 1:** Potential mechanism of pMV concentration decrease

	References	Possible mechanism of ↓ pMV concentration
ASA	[9, 18]	COX-1 inhibition
ADP receptor inhibitors	[18–21]	P2Y_12_ receptor inhibition, increase intraplatelet concentration of cAMP
PDE inhibitors	[22–24]	PDE3/PDE5 inhibition, increase cAMP concentration
GP IIb/IIIa antagonists	[25–28]	GP IIb/IIIa inhibition
Heparin	[29]	Mediation of an attractive interaction between phospholipid membranes
Statins	[30–32]	Rho-kinase pathway, activation of PPARs, reduction of NF-κB activity
Fibrates	[33, 34]	Activation of PPAR-α, increase cAMP and cGMP concentration, COX-1 inhibition, inhibition of Ca^2+^ concentration
PUFAs	[35–37]	Substrates for COX and competition with AA, incorporation into the phospholid cell membrane
Hypoglycemic agents	[38–40]	Adiponectin-dependent and NO-dependent pathway
Calcium channel blockers	[7, 41–44]	Calcium influx inhibition and decrease intracellular calcium concentration, PPAR activation
Low-calorie diet	[14, 45]	Decrease level of leptin

*AA* arachidonic acid, *ADP* adenosine diphosphate, *ASA* acetylsalicylic acid, *cAMP* cyclic adenosine monophosphate, *COX* cyclooxygenase, *NF-κB* nuclear factor kappa B, *NO* nitric oxide, *PDE* phosphodiesterase, *pMV* platelet-derived microvesicles, *PPAR* peroxisome proliferator-activated receptor, *PUFAs* polyunsaturated fatty acids

## Platelet-Derived Microvesicles

### Release of Platelet-Derived Microparticles

The blebbing of pMV is triggered by platelet activation via high shear stress [46, 47], low temperature [48], hypoxia [49], oxidative stress, endotoxins, and binding of agonists to the membrane receptor [50]. Platelet activation results in signal transduction across the cell membrane, opening of calcium channels, mobilization of calcium ions, and increase in intracellular calcium concentration [51]. It is the principal step in MV formation, leading to activation of several calcium-dependent enzymes and resulting in alteration in the lipid bilayer, loss of membrane phospholipid asymmetry, and externalization of negatively charged phospholipids, mostly phosphatidylserine (PS). Moreover, microparticle blebbing requires degradation and reorganization of cytoskeletal proteins depending mainly on calpains—cytosolic cysteine proteases—that activate integrins and disintegrate structural proteins, including actin-binding protein, talin, and the heavy chain of myosin. Moreover, gelsolin, an enzyme specific to platelets only, decomposes the capping proteins at the ends of the actin filaments. In contrast, the release of apoptotic microparticles depends mainly on activation of caspase 3 as well as Rho-associated kinase (ROCK). Their activation also leads to cytoskeletal modifications resulting in membrane blebbing [52]. Moreover, the release of MV from resting platelets is calcium and calpain independent, and it is associated with αIIβ3 integrin-mediated actin cytoskeleton destabilization [53].

### Properties of Platelet-Derived Microvesicles

Platelet-derived microvesicles participate in reactions as platelets do, since they expose various receptors also present on the platelet surface, including integrin glycoprotein (GP) such as GP IIb/IIIa (CD41/CD61), GP IX (CD42a), and GP Ibα (CD42b) [54], as well as CD40L [55] and P-selectin (CD62P) [4, 55, 56]. Ex vivo studies suggest that receptor composition depends on the physiological agonists used to activate platelet vesiculation [57]. However, some of the circulating vesicles exposing typical platelet receptors such as GP IIb/IIIa and containing full-length filamin A are in fact derived from megakaryocytes, and only those vesicles exposing platelet activation markers such as P-selectin, lysosome-associated membrane protein-1 (LAMP-1), and immunoreceptor-based activation motif receptors are considered truly derived from activated platelets [58, 59]. Platelet-derived microvesicles also contain many other factors involved in thrombosis, angiogenesis, and inflammation, including platelet-activating factor (PAF) [60], vascular endothelial growth factor (VEGF) [61], β-amyloid protein precursor [62], anticoagulant protein C/S [63], complement C56b-9, arachidonic acid (AA) [64], and chemokines [65]. Therefore, they exhibit a wide range of activities that are often opposed, including procoagulant as well as anticoagulant, proinflammatory, proatherogenic, and immunomodulatory. Platelet microvesicles participate in various processes such as intercellular communication, atherosclerosis, tissue regeneration, and tumor metastasis. Microvesicles of platelet origin account for approximately 25% of the procoagulant activity in blood [63], and their surface exhibits 50- to 100-fold higher procoagulant activity than the surface of activated platelets [66]. This procoagulant effect associated with exposure on their surface of negatively charged phospholipids lasts longer than that caused by activated platelets and is exerted distant from the site of platelet activation [67]. Platelet-derived PS+ microvesicles possess high-affinity binding sites for activated coagulation factors such as factors IXa, Va, Xa, and VIII, providing the background for thrombin formation [68–70]. On the other hand, pMV also exhibits anticoagulant activities by facilitating inactivation of factors Va and VIIIa by activated protein C [63]. The participation of pMV in angiogenesis involves the promotion of endothelial cell (EC) migration, survival, and tube formation as well as stimulation of smooth muscle cell proliferation [16]. Platelet-derived microvesicles also supply AA to ECs and induce endothelial production of cyclooxygenase-2 (COX-2) and vasoconstricting thromboxane A2 (TXA2) [71, 72]. Since pMV contains the proinflammatory chemokine RANTES and exposes GPIb—a platelet ligand for leukocyte integrin Mac-1 (CD11b/CD18, αMβ2)—they are involved in pathogenesis of inflammation and facilitate tethering of monocytes to the activated endothelial cells [64, 72, 73]. Moreover, binding of P-selectin-positive pMV to leukocytes via P-selectin-glycoprotein ligand-1 (PGSL-1) results in leukocyte, especially monocyte, activation and rolling [74]. Platelet-derived microvesicles deliver CD154 (CD40L) and thus stimulate B cell production of antigen-specific immunoglobulin G and modulate the adaptive immune response via CD4+ cells [75]. Platelet MVs are also involved in tumor growth and metastasis as they promote angiogenesis and transfer GP IIb/IIIa to cancer cells; thus, they facilitate adhesion of cancer cells to fibrinogen and endothelial cells [75]. Platelet microvesicles, as important intercellular carriers of ribonucleic acids such as messenger RNAs (mRNAs) and microRNAs (miRNAs), are able to transfer genetic information and induce changes of gene expression in recipient cells [76].

### Methods of pMV Measurement

Recently, remarkable progress has occurred in the methods of MV detection. Although conventional flow cytometry (FCM) currently remains the most widely used, it is still an imperfect method to identify different subtypes of MV based on their size as well as the presence of specific antigens on their surface using monoclonal antibodies. Some novel techniques, such as nanoparticle tracking analysis, transmission electron microscopy, tuneable resistive pulse sensing, dynamic light scattering, and high-sensitivity or impedance-based FCM, are being introduced to microparticle detection. It should be noted that perfect methods of MV measurement would provide insight not only into cellular origin but also into biochemical properties because both quantitative and qualitative analyses of MV are essential. In the identification of pMV, multiple factors including appropriate blood sample collection (size of the needle used to obtain a blood sample, type of anticoagulant, avoidance of placement of a tourniquet), centrifugation steps during preparation of plasma samples, and conditions of sample storage are important to avoid unwitting platelet activation and heterogeneity of results. The results of MV measurements available in the literature differ significantly because of the variability in pre-analytical conditions, as well as the lack of standardization of the methods [77]. Therefore, wider application of pMV measurement in both the diagnosis and the prognosis of cardiovascular risk, as well as cardiovascular therapy monitoring, is still confined. Hence, it is very important to unify the procedures and protocols, improve the comparison of measurements between the laboratories, and increase sensitivity of flow cytometry. Recently, some initial efforts have been made to standardize the pre-analytical and analytical procedures and improve sensitivity as well as reproducibility of MV detection techniques, such as using size-calibrated fluorescent beads, forward scatter parameter resolution improvement, and reduction of background noise. Platelet-derived microvesicles are the most sensitive to pre-analytical variables; therefore, the International Society on Thrombosis and Hemostasis (ISTH) Vascular Biology Standardization Subcommittee (VB SCC) has organized a first collaborative workshop aimed at standardizing pMV measurement by flow cytometry [78].

## The Impact of Pharmacological Modulation on Platelet-Derived Microvesicles

### Antiplatelet Therapy

#### Irreversible Cyclooxygenase Inhibitors

Antiplatelet agents are widely used in both prevention and treatment of vascular disease, but their impact on pMV release is still poorly understood. Acetylsalicylic acid (ASA) is an irreversible inhibitor of the isoenzymes COX-1 and COX-2; thus, it inhibits production of direct prostaglandin (PG) precursors and consequently other prostanoids, prostacyclin (PGI2) and thromboxane A2 (TXA2), from AA. The inhibition of COX-2 is related to the anti-inflammatory effect of ASA, while approximately 170-fold greater inhibition of COX-1 is responsible for its antithrombotic action [79]. Moreover, ASA initiates formation of anti-inflammatory lipoxins that inhibit proinflammatory processes such as production of tumor necrosis factor alpha (TNF-α) in T lymphocytes and activation of the transcription nuclear factor-kappa B (NF-κB) in monocytes and macrophages. Platelet-derived microvesicles transfer AA—a strong platelet agonist—between platelets, ECs, and monocytes and hence modulate their function [64, 71]. Microvesicles also metabolize AA to TXA2 and further to its stable form—TXB2 [71]. In human monocytoid cells exposed in vitro to pMV-derived AA, expression of COX-2 and eicosanoid synthesis in a PKC/p42/p44 MAPK/p38 kinase-dependent manner were observed [80]. Moreover, pMV-derived AA stimulates transcriptional activity of COX-2 as well as c-Jun and Elk-1 proteins, induces chemotaxis of monocytes, and upregulates intracellular cell adhesion molecule-1 (ICAM-1); thus, it enhanced adhesion of monocytes to ECs in vitro [72, 80]. Not only does pMV facilitate transcellular delivery of AA but also endothelially derived AA is transferred to pMV and further metabolized to TXA2 [71].

The reports on responsiveness of pMV to treatment with ASA in humans are limited and contradictory. It is worth emphasizing that the available study results are different in terms of the microparticle subtype being evaluated and the methodology that was used. Acetylsalicylic acid reduces pMV production in healthy subjects [18] as well as in patients with hypertensive heart disease and non-significant (< 50% lumen narrowing) coronary artery disease [9]. Despite this, the vast majority of reports suggest a limited impact of ASA on pMV formation. Acetylsalicylic acid does not significantly affect the release of pMV in either the acute or the chronic phase of ischemic stroke, in patients with multi-infarct dementia, in patients with non-valvular atrial fibrillation (AF) without any previously antithrombotic therapy, or in those in whom treatment was changed from ASA to warfarin [6–8, 81]. No substantial difference in the percentage of pMV was found in patients with type 1 or type 2 diabetes before and after use with a low dose of ASA [82, 83], or between diabetic patients treated with aspirin and those who were not undergoing this therapy [13]. Likewise, treatment with ASA also did not modify pMV concentration in patients after an acute coronary syndrome (ACS) [41] in stable CAD patients [84] or those who had undergone coronary angioplasty [85]. Acetylsalic acid therapy had no effect on pMV exposure of tissue factor (TF), P-selectin, or GPIIIa in patients with peripheral arterial occlusive disease (PAOD) [86] and did not affect either the release or the exposure of TF or PS on microvesicles in healthy subjects [87]. These contradictory reports may be the result of different duration of the treatment, dose of ASA, relatively small study groups, and different methodologies used for MV identification and quantification. The lack of effect of ASA on pMV release may also be associated with its insufficient drug serum level, especially since compliance in taking ASA was objectively assessed only in two of the above-cited studies [82, 86]. Moreover, a low dose of ASA might not be sufficient and strong enough to inhibit microvesiculation [87]. In addition, pMV concentrations in healthy subjects are lower than in those suffering from cardiovascular disease; therefore, the effect of antiplatelet agents in this group on MV release may not be as pronounced as in populations with cardiovascular diseases. Further studies are required to assess whether an increased dose of ASA or combination of antiplatelet therapy can reduce pMV formation. It is also necessary to explore the possible role of pMV as a biomarker in monitoring individual response to antiplatelet therapy and to explore the relationship between “ASA resistance” and pMV release.

#### Adenosine Diphosphate Receptor Inhibitors

P2Y_12_ ADP receptor is coupled with the G_i_ protein and adenylyl cyclase pathway, contributing to activation of the GP IIb/IIIa receptor for fibrinogen, which plays an important role in platelet vesiculation [49]. P2Y12 receptor antagonists, through increasing the intraplatelet concentration of cyclic adenosine monophosphate (cAMP), decrease platelet sensitivity to activation and the subsequent platelet vesiculation. The decrease in pMV formation was confirmed in healthy subjects after use of clopidogrel, a thienopyridine class agent irreversibly inhibiting platelet P2Y_12_ ADP receptor [18], and in patients with ACS treated with clopidogrel and subcutaneous low-molecular-weight heparin [41]. A negative correlation between clopidogrel plasma concentration and pMV release in stable CAD [19], as well as elevated percentage of circulating pMV related to high platelet reactivity on clopidogrel therapy in patients with ACS [88] and in those treated with percutaneous coronary intervention (PCI) [20], confirms that pMVs are potential markers of the antiplatelet effect of clopidogrel.

The active metabolite of prasugrel, a newer thienopyridine P2Y_12_ receptor antagonist, as well as the first intravenous antagonist cangrelor, have also been shown to inhibit pMV release in vitro [89–91]. According to our best knowledge, to date, there has been no more research showing the influence of other ADP-P2Y_12_ receptor inhibitors, such as ticagrelor or elinogrel, on platelet-derived microvesicles.

#### Phosphodiesterase Inhibitors

Cilostazol is a selective reversible inhibitor of phosphodiesterase type 3 (PDE3) that increases the concentration of cAMP in platelets by inhibiting adenosine uptake. It results in inhibition of platelet aggregation related to the cAMP-dependent increase in the active form of protein kinase A (PKA) in platelets. Moreover, increased cAMP concentration leads to an influx of free calcium ions back into platelet granules, which may have an important role in reducing pMV release. In addition, cilostazol has been shown to increase nitric oxide (NO) production in interleukin (IL) 1b-stimulated vascular smooth muscle cells, resulting in platelet inhibition [92]. Nonetheless, there are conflicting data on the impact of treatment with cilostazol on pMV formation, probably resulting from the varying methodology for determining platelet-derived microvesicles. The level of pMV determined by an enzyme-linked immunosorbent assay (ELISA) did not decrease after antiplatelet therapy with cilostazol in the chronic phase of cerebral infarction [8]; however, studies using flow cytometry for pMV detection reported reduced formation of pMV in arteriosclerosis obliterans [93] and diabetes [22, 23]. The concentration of circulating pMV was also decreased after combined antiplatelet therapy with cilostazol and ASA in acute ischemic stroke patients with either small or large artery occlusion [24].

Dipyridamole is another PDE3 inhibitor that inhibits cyclic guanine nucleotide degradation by PDE5 in platelets, thus elevating intracellular cyclic guanosine monophosphate (cGMP) and stimulating the release of endothelial PGI2 as well as nitric oxide. Because dipyridamole elevates adenosine and subsequent intraplatelet cAMP, and cilostazol inhibits cAMP breakdown, dipyridamole potentiates the antiplatelet effect from cilostazol [93, 94]. Indeed, in patients with arteriosclerosis obliterans, combined therapy with cilostazol and dipyridamole reduced the number of pMV compared to treatment with cilostazol alone [93]. Concomitant initial treatment with cilostazol and prednisolone in a patient with active idiopathic thrombocytopenic purpura (ITP) and coexisting acute ischemic stroke resulted in a decrease of pMV count. However, in ITP, the effect of steroids appear to be crucial for affecting the activation status of platelets and decreased pMV release, since the reduction of the steroid dose triggered a re-increase of circulating pMV [95].

#### GP IIb/IIIa Antagonists

GP IIb/IIIa is the most abundant platelet membrane receptor (approximately 80,000 copies per platelet) [96] and plays a major role in the regulation of platelet aggregation. GP IIb/IIIa on resting platelets is maintained in an inactive form and serves as a low-affinity adhesion receptor for fibrinogen; however, during platelet activation, a conformational change at the GP IIb/IIIa receptor site allows binding not only to fibrinogen but also to von Willebrand factor, thrombospondin, fibronectin, and vitronectin [97]. Monoclonal antibodies against the GP IIb/IIIa receptor inhibit release of pMV, and the ability to form pMV is severely impaired in patients with Glanzmann’s thrombasthenia [49], an inherited platelet disorder of qualitative or quantitative deficiencies in the GP IIb/IIIa receptor. This all confirms that the GP IIb/IIIa complex plays an important role in platelet blebbing. Moreover, the breakdown of the GP IIb/IIIa complex by calcium chelation abolishes MV blebbing in response to platelet activation with collagen [49]. Since GP IIb/IIIa is crucial for pMV release, dedicated inhibitors appear to be a promising group of drugs affecting MV formation. This has been proven in several in vitro studies with different GP IIb/IIIa antagonists [25, 49, 98, 99]. The release of pMV was also completely abolished by abciximab in healthy donors [26]. There was also a significant decrease of circulating PS+ procoagulant pMV in patients with ST elevation myocardial infarction (STEMI) treated with abciximab after primary PCI compared to patients who did not receive this drug. However, this effect on pMV was not found for another GP IIb/IIIa antagonist, eptifibatide, presumably due to its shorter half-life and reduced affinity for the receptor [25, 27]. Despite this, in patients with NSTEMI, eptifibatide in combined therapy with ASA, enoxaparin, and clopidogrel resulted in a significant fall in the formation of pMV [28]. The lower percentage of pMV observed in GP IIb/IIIa inhibitor-treated patients points to platelet shedding as a possible target of this drug in patients who also received a standard dual antiplatelet therapy with ASA and clopidogrel. Analyzing the effectiveness of PCI on pMV formation is related to difficulties in separating the effect of the invasive procedure from the impact of drugs usually administered during intervention. However, in patients undergoing elective PCI, abciximab reduced periprocedural increase in PMV concentrations, which was not observed among PCI patients not receiving this treatment [100]. Moreover, after bare metal stent (BMS) implantation, the pMV concentration significantly increased compared to diagnostic catheterization alone [101]. Moreover, months after the implantation of coronary stents, the pMV count was significantly higher in the drug-eluting stent (DES) group compared to the BMS group [102].

#### Other Antiplatelet Agents

A synthetic analog of PGI2—epoprostenol—is a vasodilator and platelet aggregation inhibitor clinically used for the treatment of pulmonary arterial hypertension. Platelet aggregation is inhibited through c-AMP-dependent protein kinase and phosphorylation of vasodilator-stimulated phosphoprotein (VASP). A study conducted in vitro revealed that epoprostenol almost completely inhibits pMV formation [103].

There is no study addressing the effects of new classes of antiplatelet agents, such as vorapaxar (a thrombin protease-activated receptor-1 (PAR-1) antagonist) or the thromboxane receptor antagonist terutroban, on pMV formation. However, pMVs are a promising new target for these drugs and deserve further research.

### Anticoagulants

#### Heparins

In the course of heparin-induced thrombocytopenia (HIT), binding of heparin-dependent immunoglobulin G to platelet FcγRIIa receptor (CD32a) led to platelet activation and the generation of procoagulant pMV associated with thrombotic complications [104]. Therefore, when HIT is suspected, heparin should be discontinued and an alternative anticoagulant agent should be considered to prevent thromboembolic events. Nevertheless, long-term anticoagulant therapy with the low-molecular-weight heparin (LMWH) tinzaparin led to a decrease in the procoagulant activity of circulating MV in patients with deep venous thrombosis (DVT). This effect was not observed in patients with DVT initially treated with vitamin K antagonists such as acenocoumarol, and even an increase of procoagulant activity of MV was observed in this group of patients [105].

More attention has been paid recently to the beneficial role of heparin in cancer and metastases [106]. One of its anticoagulant as well as antineoplastic mechanisms is the mediation of the release of extracellular MV, including that of platelet origin. Heparins suppress microvesiculation by mediating an attractive interaction between phospholipid membranes and thereby also act as antitumor agents [29]. Therapeutic doses of nadroparin added to the blood samples of healthy donors and patients with gastrointestinal cancer and rheumatoid arthritis suppressed microvesiculation [29]. Furthermore, heparin has also been shown to block transfer of MV and hence derived contents such as proteins, RNA, and miRNA into recipient cells. Such mechanisms of heparins’ action may be considered in the context of therapeutic strategies for the treatment of vascular diseases in which MVs play an important role.

#### Vitamin K Antagonist

In patients with non-valvular AF without any previous antithrombotic therapy, initiation of oral anticoagulation with warfarin (the target range for the international normalized ratio was 2 to 3) did not significantly affect the release of platelet-derived microvesicles [81]. Moreover, there were no significant differences in pMV concentration among AF patients who were receiving ASA and warfarin. In the AF patients in whom antithrombotic treatment was changed from ASA to warfarin, there was also no significant effect on pMV release [81]. As mentioned above, decrease in the procoagulant activity of circulating MV was also not observed in patients with DVT treated with the vitamin K antagonist acenocoumarol, and even an increase of this activity was observed [105].

#### Others

Bivalirudin, a specific and reversible direct thrombin inhibitor (DTI), administered periprocedurally during PCI in a patient with CAD, compared to unfractionated heparin (UFH), reduced the thrombogenicity of pMV measured by TF activity of pMV stimulated with TRAP or ADP [107].

Platelet-derived microvesicles have been demonstrated to express factor Xa activity at their surface, and hence, they possess significant procoagulant activity [108]. The inhibiting effect of factor Xa inhibitors on the prothrombinase activity of pMV both in vitro and in vivo was observed in an animal model [109]. However, direct inhibitors of factor Xa, such as apixaban and rivaroxaban, did not affect platelet activation after stimulation with different agonists [110]; thus, their impact on pMV blebbing is probably negligible. Currently, no data are available on the effect of novel anticoagulants, such as direct inhibitors of factor Xa (fondaparinux, rivaroxaban, apixaban, or edoxaban) and the thrombin inhibitor dabigatran, on pMV formation.

### Hypolipemic Therapy

#### HMG-CoA Reductase Inhibitors (Statins)

In addition to the well-recognized lipid-lowering effect, the pleiotropic effect of 3-hydroxy-3-methylglutaryl-coenzyme A (HMG-CoA) reductase inhibitors (statins) includes endothelial function improvement, reduction of thrombin generation, atherosclerotic plaque stabilization, decrease in expression, and activity of many inflammatory agents and metalloproteinases. Increasing evidence indicates that statins limit platelet activation and aggregation. Modifying G protein functions and the Rho kinase pathway, both involved in cytoskeleton reorganization, statins affect pMV shedding, decreasing the number of MV in a lipid-independent manner [111, 112]. Moreover, statins significantly reduce the surface exposure of proinflammatory CD40L on platelets [113] as well as on pMV [114].

HMG-CoA reductase inhibitors contribute to reduced NF-κB activity and increased exposure of peroxisome proliferator-activated receptors (PPARs); thus, they affect the release of many proinflammatory cytokines and chemokines [115], thereby limiting platelet activation.

Hypercholesterolemic patients treated with simvastatin, atorvastatin, or rosuvastatin have lower pMV concentrations compared to untreated patients with the same plasma lipid levels [111]. The percentage of pMV was also decreased after therapy with simvastatin in hyperlipidemic patients after ischemic stroke [30]. Withdrawal of rosuvastatin in patients with coronary heart disease (CHD) caused an increase in the formation of circulating pMV despite continuation of treatment with clopidogrel [31]. Combined therapy with simvastatin and losartan reduced pMV concentrations in patients with hypertension and type 2 diabetes as well [114]. Pitavastatin alone did not decrease pMV in hyperlipidemic, diabetic patients; however, the addition of eicosapentaenoic acid (EPA) significantly lowered the proportion of pMV, and the reduction was even greater than that observed with EPA alone [35]. Thus, pitavastatin enhances the antiplatelet effect of EPA in an adiponectin-dependent pathway. In patients with PAOD as well as diabetes with dyslipidemia, combined treatment with atorvastatin and ASA significantly reduced TF, P-selectin, and GPIIIa exposure on pMV, while the exposure of PS was not influenced by atorvastatin [86, 116]. Lipid-lowering treatment with simvastatin reduced the total count of procoagulant pMV as well as the exposure of P-selectin, CD40L, and TF on pMV in diabetic patients with chronic kidney disease. However, in diabetic patients without kidney disease, a reduction of pMV percentage was not achieved until combined therapy with simvastatin and ezetimibe was applied [32]. This suggests that in patients with diabetes and concomitant kidney disease, increased platelet activation is observed and pMVs are involved in the hypercoagulable state in chronic kidney disease. Treatment with pravastatin significantly reduced the GPIIIa receptor for fibrinogen on pMV in type 2 diabetes patients with no effect on the number of pMV or the exposure of TF antigen [117].

#### Fibrates

Bezafibrate is an agonist of PPAR-alpha, which are also present in human platelets and are involved in many biological functions such as inflammation, atherosclerotic plaque formation, and lipid metabolism. Activation of PPARs inhibits platelet function via inhibition of PKCα and increases the levels of both cAMP and cGMP [118, 119]. The antiplatelet effect of PPAR agonists is also associated with direct inhibition of COX-1 [120] as well as indirect inhibition of intracellular Ca^2+^ mobilization, TXA2 production, exposure of P-selectin, and pMV release. Decreased formation of pMV is presumably caused by reduced calcium concentration, inhibition of PKC, and activation of PKA via increased concentration of cAMP [33].

The proportion of pMV was significantly decreased after treatment with bezafibrate in patients with connective tissue diseases and secondary hyperlipidemia caused by long-term steroid administration, as well as in patients with diabetes without obstructive CAD burdened with elevated levels of remnant-like particle cholesterol (RLP-C) [33, 34]. RLP-C in vitro increases intracellular oxidative stress, reduces NO, and affects endothelial-dependent vasorelaxation, thus inducing platelet activation. Therefore, RLP-C may be a target, since a decrease of its elevated level also results in the reduction of pMV formation and lower incidence of cardiovascular events in patients with diabetes [34].

#### Inhibitors of Cholesterol Intestinal Absorption

Ezetimibe selectively inhibits the intestinal absorption of cholesterol without inhibition of cholesterol synthesis in the liver or increase of bile acid excretion [121]. Treatment with ezetimibe showed no effect on pMV either in subjects with stable CHD or in those with CHD risk factors receiving double therapy with simvastatin and ezetimibe [84, 122]. Moreover, it was demonstrated that in diabetic patients with chronic kidney disease, combined therapy with ezetimibe and simvastatin had no further effect on pMV formation compared to simvastatin therapy alone [32].

Although preliminary reports suggested that shedding of pMV is the result of changes in cholesterol content within platelet membranes [123], the lack of association between cholesterol concentration and glycoprotein exposure on pMV refutes that mechanism [86].

#### Other Hypolipidemic Treatment

In patients with familial hypercholesterolemia, the procedure of lipoprotein apheresis not only removed LDL-cholesterol but also reduced the concentration of MV, although the percentage of annexin V-positive MV, including those of platelet origin, did not change [124]. There is no study addressing the effects of other hypolipidemic agents such as niacin and bile acid sequestrants or new promising agents such as proprotein convertase subtilisin/kexin type 9 (PCSK9) inhibitors or cholesterylester transfer protein (CETP) inhibitors on pMV, and further studies are needed.

### Hypoglycemic Therapy

Platelet-derived microvesicles are elevated in diabetes and play an important role in the development of atherosclerotic vascular complications in this condition [125]. Postprandial hyperglycemia resulting in decrease in NO production is thought to be a significant factor of platelet activation in diabetes [38]. The reports on the effects of hypoglycemic agents on the release and procoagulant activity of pMV are limited to α-glucosidase inhibitors, teneligliptin and mitiglinide. Alpha-glucosidase inhibitors mainly reduce postprandial hyperglycemia and hyperinsulinemia via inhibition of the absorption of carbohydrates from the intestine. Gliptins such as teneligliptin are dipeptidyl peptidase-4 (DPP-4) inhibitors that increase concentrations of active incretin hormones such as gut-derived glucagon-like peptide-1 (GLP-1) and glucose-dependent insulinotropic peptide. It leads to the stimulation of insulin release and inhibition of glucagon release and therefore decreases blood glucose. Presumably, the antiplatelet effects of these drugs, including the reduction of pMV release, are adiponectin and NO dependent [39]. Low NO concentration related to hypoadiponectinemia and postprandial hyperglycemia results in platelet activation [126]. Therefore, increased concentrations of adiponectin caused by mitiglinide exert an antiplatelet effect by promoting NO production, and improvement of hypoadiponectinemia may result from decreased pMV formation [127]. Moreover, therapy with both α-glucosidase inhibitors and gliptins enhances active GLP-1 secretion, which subsequently promotes adiponectin secretion [40, 128].

Hypoglycemic therapy with the α-glucosidase inhibitors acarbose [40] and miglitol [38] significantly decreased the percentage of pMV, which was considerably higher in diabetic patients with a previous history of thrombotic complications than in the non-thrombotic group. Administration of mitiglinide in monotherapy as well as combined therapy to diabetic patients significantly reduced plasma concentrations of pMV [127]. Teneligliptin therapy also significantly reduced the pMV number in diabetic subjects, with a more significant reduction in hemodialyzed patients than in those not dialyzed [39]. However, these subjects received teneligliptin monotherapy or teneligliptin in combination with other antidiabetic drugs, and therefore, evaluation of the therapeutic effects is difficult.

At the moment, there are no more studies addressing the effects of other hypoglycemic agents on pMV formation; however, the group of antidiabetic agents, including PPAR-gamma agonists such as pioglitazone and rosiglitazone, are promising drugs affecting MV [129, 130] and potentially also pMV release because of attenuating platelet activation.

### Antihypertensive Therapy

Increased formation of pMV in hypertension is associated with activation of platelets as a result of elevated shear force. The formation of pMV positively correlates with both systolic and diastolic blood pressure [12]. The main stage of MV release is an increase in cytoplasmic calcium ions associated with the opening of plasma membrane calcium channels. Therefore, the calcium channel blockers that inhibit calcium influx and decrease intracellular calcium concentration are promising agents hampering the process of microvesiculation. Moreover, dihydropyridine calcium channel blockers enhance NO activity and increase adiponectin concentration, which regulate platelet activation and hence microvesiculation [131]. Beneficial effects of nifedipine, a dihydropyridine calcium channel blocker and PPAR agonist, on reducing pMV formation were observed in patients with transient ischemic attacks [7] as well as in hypertensive patients with type 2 diabetes [42, 43]. A decrease in pMV number was also observed in hypertensive patients with type 2 diabetes after treatment with efonidipine, another dihydropyridine calcium channel blocker [44], and in patients with ACS treated with different calcium channel antagonists compared to those not receiving such treatment [41, 85].

The angiotensin II receptor antagonists (sartans) are able to selectively inhibit the platelet angiotensin II type 1 receptor, with the result of lower responsiveness of platelets to increased intracellular Ca^2+^ levels, and consequently inhibit MV blebbing. In monotherapy of hypertensive patients, eprosartan normalized the elevated percentage of pMV [132]. In hypertensive patients with diabetes, the percentage of pMV was also decreased during monotherapy with another angiotensin receptor blocker, losartan. Furthermore, the decrease of pMV number was greater in hypertensive and hyperlipidemic patients administered combined therapy with losartan and simvastatin than in those without type 2 diabetes [114], preventing the development of cardiovascular complications via a mechanism other than reduction of the blood pressure or lipid levels.

Spironolactone is a potassium-sparing diuretic that, among other effects, decreases intraplatelet Na^+^ and Ca^2+^ concentration by reducing activity of the Na^+^ pump [123]. In an experimental rat model of aldosterone-mediated hypertension, the diminished pMV formation observed after spironolactone treatment presumably is associated with a lower increase in intracellular Ca^2+^ in platelets [133].

### Non-Pharmacological Interventions

Dietary and life style modification are the first-line prevention and therapy for vascular diseases.

It has been proven that a diet enriched in omega-3 polyunsaturated long-chain fatty acids (PUFAs) is associated with reduced incidence of thrombotic events. Beneficial effects of PUFAs such as EPA or docosahexaenoic acid (DHA) in vascular diseases include improvement of endothelial function, inhibition of platelet aggregation, and decrease in blood pressure and plasma triglyceride concentration. Omega-3 fatty acids, as substrates for COX, compete with AA, resulting in reduced formation of proaggregatory eicosanoids. Moreover, incorporation of omega-3 PUFAs, especially EPA, into the phospholipid cell membranes leads to membrane remodeling then improves the rheological properties of blood and reduces platelet activation [134]. A single oral dose of EPA-rich oils immediately and significantly reduced the pMV procoagulant activity determined by the annexin V binding and thrombin generation in healthy individuals, probably by incorporating fatty acids into the platelet membrane. Simultaneously, there were no changes in pMV number, suggesting that the new pMVs generated following EPA supplementation were less procoagulant [134]. Combined long-lasting administration of EPA and DHA also caused normalization of TF-dependent procoagulant activity of pMV in patients after myocardial infarction, with no significant effect on the total number of microvesicles [36]. In contrast, the percentage of pMV decreased significantly in hyperlipidemic, diabetic patients receiving EPA [35, 37]. In spite of the fact that the omega-3 PUFAs are thought to exhibit presumed protective effects in CVD, there is currently a lack of strong evidence to recommend the routine use of omega-3 PUFAs in the primary and secondary prevention of cardiovascular disease [135].

Excessive adipose tissue is associated with increased formation of pMV [14], and pMV release positively correlates with increased thrombin formation in obese subjects [136]. Adipose tissue induces low-grade inflammation and produces cytokines such as TNF-α and IL-6 that enhance pMV production [21]. In obese patients, an increased level of leptin promotes ADP-induced platelet aggregation and therefore also ADP-induced pMV generation [137, 138]. Moreover, even in normal-weight acute stroke subjects, increased pMV formation positively correlated with plasma leptin concentrations [139]. Elevated plasma concentrations of oxidative stress markers, such as oxidized low-density lipoprotein (oxLDL), promote the shedding of pMV both in vitro [140] and in vivo [141].

In a rat model, a long-term high-fat diet providing 60% energy as fat generated a significant increase of total MV with a significantly elevated number of pMV [142]. A similar process was also observed after two consecutive high-fat meals in healthy men [141]. Introduction of a short-term, very-low-calorie diet in obese women resulted not only in the reduction of body weight, lowering of blood pressure, and improvement of metabolic parameters but also a significant reduction in the percentage of procoagulant pMV [45]. Body weight loss by reducing calorie restriction with as well as without aerobic physical exercise also decreases the number of pMV, probably through reduction of the amount of adipose tissue [14].

The oat is considered a low glycemic index plant, which is expected to modulate postprandial hyperglycemia and exert direct anti-inflammatory and antioxidant effects. An oat-enriched diet in patients with type 2 diabetes reduced both the concentration and the proportion of TF-positive pMV [143]. It was also demonstrated that cocoa consumption suppressed ADP-induced or epinephrine-induced platelet activation and hence pMV formation. The proportion of MV detected by flow cytometry was reduced up to 6 h after consumption of the beverage cocoa, unlike the formation of MV after consumption of a caffeine-containing beverage, which was higher than baseline [143].

Red wine polyphenols have beneficial properties for preventing cardiovascular disorders by their influence on NO balance and prevention of oxidative stress. In an experimental rat model of aldosterone-mediated hypertension, red wine polyphenols also significantly reduced pMV formation [133]. Interestingly, preconsumption of red wine prevented most of the acute effects of cigarette smoking, including reduced concentration of IL-6 protein and pMV increase [144].

Cigarette smoking is considered one of the major risk factors for atherothrombotic disease, including ischemic heart disease and stroke, and increases the risk of cardiovascular mortality. Active smoking even of one cigarette caused an immediate and significant increase in the number of circulating pMV, with a significant increase in pMV exposing CD62P and CD154 on their surface in healthy volunteers, most likely due to cell activation in response to cigarette smoke [145].

## Summary

Platelet-derived microvesicles exhibit a multitude of properties and participate in several pathological conditions. Researchers have started to pay attention to MV as novel biomarkers in vascular diseases, especially in cardiovascular risk stratification. The influence of pharmacological agents on circulating pMV may become a new therapeutic challenge in the treatment of many vascular diseases. Not only modulation of pMV release but also exposure of their surface markers could have significant therapeutic application to monitor the efficacy of antiplatelet therapy. This modulation has resulted in a series of studies dedicated to the inhibition of MV release, especially those of platelet origin (Table 2). The different methodology of isolation of pMV, as well as the use of different quantification methods, the unequal doses of studied drugs, small study groups, or different treatment duration, makes the comparison between available studies difficult. Moreover, the lack of standardized methodology resulted for a long time in overestimation of the fraction of circulating pMV, and exposure of PS or TF on pMV is often an artifact caused by non-specific antibody binding in flow cytometric analysis.

**Table 2:** The impact of vascular disease treatment on platelet-derived microvesicles

	Population/disease	Treatment strategy/dose and duration	Effect on pMV concentration	Reference	Supplementary information
COX inhibitors: ASA	Healthy subjects	100 mg/day for 3 or 7 days	↓	[18]	↓ ADP-induced pMV formation
	Healthy volunteers	100 mg/day for 7 days	No effect	[87]	
	Hypertensive heart disease and non-significant (< 50% lumen narrowing) CAD	Chronic treatment (8 weeks) with a dose of 100 mg/day	↓	[9]	
	Acute ischemic stroke	No data available	No effect	[6]	SP
	TIA, ischemic stroke, multi-infarct dementia	No data available	No effect	[7]	SP
	Chronic phase of ischemic stroke	100 mg/day for 4 weeks	No effect	[8]	SP
	AF	150 mg/day for 4 weeks	No effect	[81]	PP
	Diabetes	100 mg/day for 10 or 15 days	No effect	[82, 83]	PP
	Patients after ACS	75 mg/day for 6 months	No effect	[41]	SP
	PAOD and hypercholesterolemia	320 mg/day for 8 weeks	No effect	[86]	PP
	Diabetes	No data available	No effect	[13]	
	Stable CAD	100 mg/day for 1 week	No effect	[84]	
	CAD patients undergoing coronary angioplasty	No data available	No effect	[85]	
ADP receptor inhibitors	Healthy subjects	75 mg/day clopidogrel for 3 days	↓	[18]	↓ ADP-induced pMV formation
	Healthy volunteers	Administration of a loading dose (60 mg) of prasugrel	↓	[86]	in vitro study
	Stable CAD	75 mg/day clopidogrel for 3 weeks	↓	[19]	Negative correlation between clopidogrel plasma concentration and pMV release
	ACS patients	Clopidogrel (loading dose of 600 mg followed by a maintenance dose of 75 mg/day) for 30 days	↓	[88]	high-on clopidogrel platelet reactivity associated with higher pMV concentration SP
	ACS patients	Clopidogrel and subcutaneous LMWH (i.e., enoxaparin 1 mg per kg body weight twice daily)	↓	[41]	SP
	ACS treated with PCI	Oral loading dose of ASA (500 mg) and clopidogrel (600 mg), then DAPT (75 mg/day ASA and 75 mg/day clopidogrel) for 12 months	↓	[20]	pMV concentration higher in patients with high platelet reactivity (assessed by impedance aggregometry); SP
PDE inhibitors	Chronic phase of ischemic stroke	200 mg/day cilostazol for 4 weeks	No effect	[8]	SP
	Arteriosclerosis obliterans	100 mg/day cilostazol for 2 weeks or combined therapy with cilostazol (100 mg/day) and dipyridamole (150 mg/day) for 14 weeks	↓	[93]	↓ pMV number (more significant on combined therapy)
	Non-insulin-dependent diabetes	150 mg/day cilostazol for 4 weeks	↓	[22]	
	Diabetes with nephropathy	150 mg cilostazol	↓	[23]	
	Acute ischemic stroke	combined therapy with cilostazol (200 mg/day) and ASA (100 mg/day) for 4 weeks	↓	[24]	SP
	ITP patient with ischemic stroke	Combined therapy with 200 mg/day cilostazol and prednisolone (30 mg/day) for 30 days	↓	[95]	Elevation of pMV concentration after decrease in prednisolone dose
		Combined therapy with dexamethasone (40 mg/day for 4 days), prednisolone (30 mg/day), and cyclosporine (250 mg/day) for 1 month	↓		Revision of immunosupressive therapy resulted in normalization of plasma pMV SP
GP IIb/IIIa inhibitors	Medication-free normal volunteers	Abciximab (10 μg/mL, approximately 0.2 μm)	↓	[25]	In vitro study
	Healthy donors	Abciximab	↓	[26]	
	Medication-free normal volunteers	0.5 μm tirofiban or 0.5 μm eptifibatide	No effect	[25]	In vitro study No effect as a result of eptifibatide shorter half-life and reduced affinity for the receptor
	STEMI after PCI	Abciximab (one 250 μg/kg bolus followed by 0.125 μg/kg/min continuous infusion up to 12 h) plus standard DAPT (ASA + clopidogrel)	↓	[27]	SP
	STEMI after PCI	Eptifibatide (one 180 μg/kg bolus followed by 2 μg/kg/min continuous infusion up to 18 h) plus standard DATP (ASA + clopidogrel)	No effect	[27]	No effect as a result of eptifibatide shorter half life and reduced affinity for the receptor SP
	NSTEMI	Eptifibatide (180 μg/kg IV bolus, followed by an infusion of 2 μg/kg per min) given in addition to ASA (500 mg bolus IV followed by 75 mg/day orally), enoxaparin (1 mg/kg twice a day subcutaneously), and clopidogrel (oral loading dose 300 mg)	↓	[28]	SP
Anticoagulants	DVT	Long-term (for 3 months) anticoagulant therapy with a LMWH-tinzaparin (175 IU/kg per day)	↓	[105]	The decrease of procoagulant activity of MV
	DVT	Vitamin K antagonist—acenocoumarol for 3 months	↑	[105]	The increase of procoagulant activity of MV
	Non-valvular AF	Oral anticoagulation with vitamin K antagonist, warfarin (with the target range for INR, 2 to 3)	No effect	[81]	PP
Statins	PAOD	Combined therapy with atorvastatin (80 mg/day) and ASA (320 mg/day) for 8 weeks	↓ Exposition of TF, P-selectin, GPIIIa on pMVs	[86]	
	Hypercholesterolemic patients	Different statins: simvastatin (20 mg/day), atorvastatin (20 mg/day), or rosuvastatin (10 mg/day)	↓	[111]	Compare to untreated patients with the same plasma lipid level PP
	Hypertensive and hyperlipidemic patients with type 2 diabetes	Combined therapy simvastatin (10 mg/day) and losartan (50 mg/day) for 24 weeks	↓	[114]	
	Hyperlipidemic patients after ischemic stroke confirmed by CT	Simvastatin 20 mg/day for 6 months	↓	[30]	SP
	CAD	Withdrawal of rosuvastatin (rosuvastatin was given at daily dose of 40 mg and clopidogrel loading dose was 300 mg, followed by 75 mg daily)	↓	[31]	Increase of pMV amount after rosuvastatin withdrawal
	Hyperlipidemic, diabetic patients	Pitavastatin 2 mg/day for 6 months	No effect	[93]	
	Hyperlipidemic, diabetic patients	Combined therapy with pitavastatin (2 mg/day) and EPA (1800 mg/day) for 6 months	↓	[93]	
	Diabetes type 1 with dyslipidemia	Atorvastatin (80 mg/day) for 2 months	↓ Exposition of surface markers	[116]	↓ Exposition of GPIIIa, P-selectin on pMVs PP
	Diabetic patients with or without chronic kidney disease	Simvastatin 40 mg/day for 8–10 weeks	↓ Exposition of surface markers	[32]	↓ P-selectin, CD40L exposition on pMVs PP
	Type 2 diabetes	Pravastatin 40 mg/day for 8 weeks	↓ Exposition of surface markers No effect on pMV concentration	[117]	↓ Exposition of GPIIIa receptor for fibrinogen on pMVs
Fibrates	Patients with connective tissue diseases and secondary hyperlipidemia caused by long-term steroid administration	6-month treatment with bezafibrate	↓	[33]	
	Patients with diabetes without obstructive CAD	400 mg/day bezafibrate for 6 weeks	↓	[34]	PP
Intestinal cholesterol absorption inhibitors	Diabetic patients with or without chronic kidney disease	Simvastatin (40 mg/day) and ezetimibe (10 mg/day) for 8–10 weeks	No effect	[32]	No further effect on pMV formation compared to simvastatin therapy alone PP
	Patients with stable CAD	10 mg/day ezetimibe for 1 week	No effect	[84]	
	Subjects with CAD risk factors receiving concomitant therapy with simvastatin and ezetimibe	10 mg/day ezetimibe for 4 weeks	No effect	[122]	PP
Omega-3 PUFA	Healthy males and females	Single dose of EPA-rich (providing 1 g EPA with an EPA/DHA ratio of 5:1) or DHA-rich (providing 1 g DHA with an EPA/DHA ratio of 1:5) oil	No effect	[134]	Newly released pMV have reduced procoagulant properties, no effect on pMV number
	Hyperlipidemic patients with type 2 diabetes	EPA 1800 mg daily for 6 months or combined therapy with pitavastatin (2 mg/day) and EPA (1800 mg/day) for 6 months	↓	[93]	Higher reduction than that observed with EPA alone
	Patients after myocardial infarction	Long-lasting (over 12 weeks) administration of EPA and DHA	↓	[36]	Normalization of both elevated concentration as well as TF-dependent procoagulant activity of pMV SP
	Hyperlipidemic patients with type 2 diabetes	EPA (1800 mg/day) for 4 weeks	↓	[37]	
Hypoglycemic therapy	Diabetes type 2 with or without hemodialysis treatment	Teneligliptin 20 mg/day for 6 months	↓	[39]	
	Patients with type 2 diabetes	Miglitol 150 mg/day for 4 months	↓	[38]	
	Diabetic patients	Acarbose 300 mg/day for 3 months	↓	[40]	
	Diabetic patients	Miglitol 30 mg/day for 3 months	↓	[127]	
Antihypertensive therapy	Patients with recurrent TIA	Nifedipine (30–60 mg) for 1–6 weeks	↓	[7]	SP
	Patients after ACS	Different calcium channel antagonists for 6 months	↓	[41]	↓ pMV number (compared to those not receiving such treatment) SP
	Hypertensive, diabetic patients	Losartan 50 mg/day for 24 weeks	↓	[114]	
	Hypertensive, hyperlipidemic patients with or without diabetes	Losartan (50 mg/day) and simvastatin (10 mg/day) for 24 weeks	↓	[114]	↓ pMV percentage (greater among those with than without type 2 diabetes)
	Hypertensive patients with diabetes type 2	Nifedipine 50 mg/day for 12 months	↓	[42]	
	Hypertensive patients with diabetes type 2	Long-acting nifedipine formulation 20 mg/day for 6 months	↓	[43]	
	Hypertensive patients with or without diabetes	Efonidipine 40 mg/day for 8 weeks	↓	[44]	
	Hypertensive patients	Eprosartan 600 mg/day	↓	[132]	
	Animal model of hypertension	Spironolactone	↓	[133]	
Non-pharmacological interventions	Healthy men	Two consecutive high-fat meals (900 kcal) at time point *t* = 0 and 4 h (breakfast and lunch); each meal consisted of 50 g of fat, of which 60% was saturated, 55 g of carbohydrates, and 30 g of protein within 15 min	↑	[141]	
	Animal model (rat)	High-fat diet (providing 60% of energy as fat) for 20 weeks	↑	[142]	↑ Total MV number significant ↑ pMV number
	Obese (BMI > 25 kg/m^2^)	Reducing calorie intake with as well as without aerobic physical exercise [mean daily caloric intake approximately 1200 kcal/day ♀, 1680 kcal/day ♂ with or without aerobic exercise 3 days per week (60 min per session)]	↓	[14]	A mean weight loss of 8 kg in moderately obese subjects (mean BMI = 27.4 kg/m^2^)
	Obese women (BMI > 30 kg/m^2^)	Short-term very-low-calorie diet (600 kcal/day for 1 month and then 1200 kcal/day during the second month)	↓	[45]	↓ Procoagulant pMV percentage
Omega-3 PUFA	Healthy males and females	Single dose of EPA-rich (providing 1 g EPA with an EPA/DHA ratio of 5:1) or DHA-rich (providing 1 g DHA with an EPA/DHA ratio of 1:5) oil	No effect	[134]	Newly released pMVs have reduced procoagulant properties, no effect on pMV number
	Hyperlipidemic patients with type 2 diabetes	EPA 1800 mg daily for 6 months or combined therapy with pitavastatin (2 mg/day) and EPA (1800 mg/day) for 6 months	↓	[93]	Higher reduction than that observed with EPA alone
	Patients after myocardial infarction	Long-lasting (over 12 weeks) administration of EPA and DHA	↓	[36]	Normalization of both elevated concentration as well as TF-dependent procoagulant activity of pMV SP
	Hyperlipidemic patients with type 2 diabetes	EPA (1800 mg/day) for 4 weeks	↓	[37]	

*ACS* acute coronary syndrome, *ADP* adenosine diphosphate, *AF* atrial fibrillation, *ASA* acetylsalicylic acid, *BMI* body mass index, *CAD* coronary artery disease, *COX* cyclooxygenase, *CT* computed tomography, *DAPT* dual antiplatelet therapy, *DHA* docosahexaenoic acid, *DVT* deep vein thrombosis, *EPA* eicosapentaenoic acid, *GP* glicoprotein, *INR* international normalized ratio, *ITP* idiopathic thrombocytopenic purpura, *IV* intravenous, *LMWH* low-molecular-weight heparin, *MV* microvesicles, *NSTEMI* non-ST elevation myocardial infarction, *PAOD* peripheral arterial occlusive disease, *PCI* percutaneous coronary intervention, *PDE* phosphodiestherase, *pMV* platelet-derived microvesicles, *PP* primary vascular disease prevention, *PUFAs* polyunsaturated fatty acids, *SP* secondary vascular disease prevention, *STEMI* ST elevation myocardial infarction, *TF* tissue factor, *TIA* transient ischemic attack

In conclusion, inhibitors of the ADP receptor and statins are the most promising drugs that limit the unfavorable impact of pMV in both primary prevention and secondary prevention of vascular disease. Promising agents also include the GP IIb/IIIa antagonists, but their use is limited to acute cardiovascular disease. Treatment with ASA, an irreversible COX inhibitor, despite convincing theoretical justification, does not influence pMV release, and its role as an anti-pMV agent seems to be limited. Although there is considerable research into the effects of vascular disease treatment on pMV, their use as both potential markers in vascular diseases and monitoring of treatment is still questionable due to the lack of comparability of results and the lack of standardized methods for microparticle determination. Further studies are required to understand how pMVs respond to treatment and to determine the potential mechanisms of drug action, effective dosages, and drug interactions in the context of prevention and treatment of vascular diseases.
